# Long-Term Culture of Rat Hippocampal Neurons at Low Density in Serum-Free Medium: Combination of the Sandwich Culture Technique with the Three-Dimensional Nanofibrous Hydrogel PuraMatrix

**DOI:** 10.1371/journal.pone.0102703

**Published:** 2014-07-17

**Authors:** Ai Kaneko, Yoshiyuki Sankai

**Affiliations:** Center for Cybernics Research, University of Tsukuba, Tsukuba, Japan; Ohio State University, United States of America

## Abstract

The primary culture of neuronal cells plays an important role in neuroscience. There has long been a need for methods enabling the long-term culture of primary neurons at low density, in defined serum-free medium. However, the lower the cell density, the more difficult it is to maintain the cells in culture. Therefore, we aimed to develop a method for long-term culture of neurons at low density, in serum-free medium, without the need for a glial feeder layer. Here, we describe the work leading to our determination of a protocol for long-term (>2 months) primary culture of rat hippocampal neurons in serum-free medium at the low density of 3×10^4^ cells/mL (8.9×10^3^ cells/cm^2^) without a glial feeder layer. Neurons were cultured on a three-dimensional nanofibrous hydrogel, PuraMatrix, and sandwiched under a coverslip to reproduce the *in* *vivo* environment, including the three-dimensional extracellular matrix, low-oxygen conditions, and exposure to concentrated paracrine factors. We examined the effects of varying PuraMatrix concentrations, the timing and presence or absence of a coverslip, the timing of neuronal isolation from embryos, cell density at plating, medium components, and changing the medium or not on parameters such as developmental pattern, cell viability, neuronal ratio, and neurite length. Using our method of combining the sandwich culture technique with PuraMatrix in Neurobasal medium/B27/L-glutamine for primary neuron culture, we achieved longer neurites (≥3,000 µm), greater cell viability (≥30%) for 2 months, and uniform culture across the wells. We also achieved an average neuronal ratio of 97%, showing a nearly pure culture of neurons without astrocytes. Our method is considerably better than techniques for the primary culture of neurons, and eliminates the need for a glial feeder layer. It also exhibits continued support for axonal elongation and synaptic activity for long periods (>6 weeks).

## Introduction

The primary culture of neuronal cells plays an important role in neuroscience, especially in studies of their differentiation, nutritional requirements, and synapse formation. The ability to culture hippocampal neurons for 3–5 weeks, to allow them to become polarized and mature, extend axons and dendrites, and form synaptic connections, would be an extremely useful tool. For research on individual neurons or subcellular components, neurons should be plated at low density and maintained with a chemically defined medium because undefined components, such as serum, make it difficult to evaluate what factors are influencing neuronal growth. There has long been a need for methods enabling the long-term culture of primary neurons at low density in defined, serum-free, medium [1], [2], [3], [4].

However, the lower the cell density, the more difficult it is to maintain the cultures of primary neurons in serum-free medium. Neuronal death at low density is caused by a lack of paracrine trophic support from adjacent neurons and glia [1]. When plated at low density (≤10^4^ cells/cm^2^), rat primary neurons from hippocampi or other brain regions typically die within days, suggesting that neuronal survival is critically dependent on their density (around 10^4^ cells/cm^2^) [5], [6], [7], [8], [9], [4], [10].

Co-culture of primary neurons with glial cells is often used to support neuronal survival [6], [7], [5], [11], [1], [3], [12]. However, much like serum, glial cells are also an undefined experimental variable. Although Neurobasal medium (Gibco, Life Technologies, Carlsbad, CA, USA) supplemented with B27 and L-glutamine is suitable for long-term culture of primary neurons at high density (≥1.6×10^4^ cells/cm^2^) [8], even these methods barely support the primary culture of neurons at low densities (≤10^4^ cells/cm^2^) for 1 month or more, which is still longer than other methods without a glial feeder layer [5], [6], [7], [1], [8], [12], [13]. At densities ≤10^4^ cells/cm^2^, cell viability or neurite bearing ratio is drastically decreased to 20–40% within 1 week after plating, whereas with a greater density, viability can be maintained at a high level (50–100%) [4], [5], [6], [7], [8], [9], [10], [14], [11], [15], [16].

*In vivo*, cells grow on a three-dimensional (3D) extracellular matrix (ECM), which provides a rough and large surface of nanofibers; hence, cells grown *in* *vitro* should also prefer to be cultured on 3D nanofibrous scaffolds [17]. Most cells in 2D culture, especially neurons, grow, react, differentiate, mature and die differently than cells *in* *vivo*. Many stem cells including induced pluripotent stem cells and embryonic stem cells can differentiate normally into neurons only in 3D culture. The 3D nanofibrous hydrogel PuraMatrix (BD Biosciences, Franklin Lakes, NJ, USA) is a peptide scaffold that self-assembles into nanofibers of 7–10 nm in diameter, similar to those comprising the ECM *in* *vivo*. A number of studies have used PuraMatrix hydrogel as a scaffold for primary neurons or the rat PC12 adrenal pheochromocytoma cell line [18], [19]. This hydrogel was found to be more biocompatible than other scaffolds, such as Matrigel, and while 100% PuraMatrix decreased the viability of human neural stem cells, 25% PuraMatrix allowed their normal differentiation [20].

Low-oxygen conditions should also be replicated *in* *vitro* because the oxygen concentration *in* *vivo* is lower than that in air [21], [22]. In an effort to experimentally replicate low-oxygen conditions, the sandwich culture method was first reported [4], and it is often used in co-culture with glial cells. For many applications and investigations, it is very important that primary neurons be cultured under conditions that resemble the *in* *vivo* environment as closely as possible, especially if the researcher is aiming to determine how neurons behave *in* *vivo*.

In the present study, we cultured rat hippocampal neurons using the sandwich culture technique in combination with PuraMatrix hydrogel to reproduce *in* *vivo*-like conditions, and we achieved long-term culture (>2 months). This is the first report of this combination, which has the potential for wide application in neuroscience research. Low-density (<10^4^ cells/cm^2^) neurons were plated on 3D PuraMatrix hydrogel, sandwiched under a coverslip, and cultured in Neurobasal medium. The neurons were exposed to concentrated paracrine factors under low-oxygen, microgradients of oxygen and other components, or different microenvironmental conditions. We assessed the viability of neurons and measured neurite length to determine the best culture conditions for replicating the *in* *vivo* situation. This determination of *in* *vivo*-like culture conditions in which neurites or axons extend over long distances may be beneficial not only to fundamental neuroscience research, but also to clinical applications in regenerative medicine because central nervous system (CNS) neurons and axons demonstrate a poor regenerative capacity. Long neurites on PuraMatrix can be considered an indicator of *in* *vivo*-like conditions, because neurite elongation is promoted by an *in* *vivo*-like collagen gel [23].

## Materials and Methods

### Ethics statements

All procedures with live animals conformed to the ethical guidelines established by the Japanese Council on Animal Care and were approved by the animal care committee of the University of Tsukuba (Permit Number: 11-324, 12-274).

### 3D hydrogel

To determine the best concentration of PuraMatrix to use in the present study, we tested three concentrations (100%, 50%, and 25%) in a preliminary experiment using neuronal-like PC12 cells. These concentrations were selected because the normal protocol for PuraMatrix recommends a concentration range of 25–100%. The longest neurites on PC12 cells and primary neurons were observed when cultured on the 25% PuraMatrix, which was therefore used for all subsequent studies.

PuraMatrix at a concentration of 25% was prepared by dilution with tissue culture water (Sigma-Aldrich, St. Louis, MO, USA) at a ratio of 1∶3. Next, 0.5 mL of 25% PuraMatrix was carefully dispensed along the sidewalls of the wells of a 12-well tissue culture-treated multiplate (353043, BD Biosciences), after which 1 mL of culture medium was carefully added to induce gelation. The multiplate was placed in a 5% CO_2_ incubator for 30 min to complete gelation. Subsequently, the medium was changed carefully two to three times over a period of 1 h to equilibrate the growth environment to physiological pH from its original pH of 2–4.

### PC12 cell culture

PC12 cells were purchased from DS Pharma Biomedical Co., Ltd. (Osaka, Japan) and maintained in RPMI 1640 medium (Wako, Osaka, Japan) supplemented with 10% horse serum (Gibco) and 5% fetal bovine serum (FBS, Equitech-Bio, Inc., Kerrville, TX, USA) in tissue culture flasks (Primaria, BD Biosciences). Cells were dissociated by pipetting and plated on 12-well multiplates with or without PuraMatrix equilibrated with culture medium as described above. Immediately or 1 day after plating, nerve growth factor (NGF, 50 ng/mL in medium; BD Biosciences) was added to the cells to induce neuron-like differentiation. Cell morphology and neurite length were observed using an inverted microscope (Ti-S, Nikon, Tokyo, Japan).

### Dissection and dissociation of primary hippocampal neurons

Hippocampal neurons were isolated from Wistar rat embryos on embryonic day 18 (E18) or E19. The hippocampi of E18–19 rats are homogenously composed of major pyramidal neurons and a few glial cells, and are generally used as a source of primary neurons [3]. Pregnant female Wistar rats were purchased from SLC (Shizuoka, Japan).

Approximately 10 fetuses were obtained from each rat, and whole brains were isolated from the fetuses. About 20 hippocampi were dissected from the brains under a stereoscopic microscope using two pairs of fine tweezers. The meninges were carefully removed from the hippocampi. All fetuses and tissues were maintained in minimum essential medium (MEM, Sigma) and chilled on ice. All subsequent procedures except for incubation and centrifugation were performed in a laminar flow hood.

After dissection, the hippocampi were washed gently with 7–8 mL of phosphate-buffered saline (PBS, Wako) in a 15-mL conical tube three times. After washing, 5 mL of a papain solution and 20–60 µL of deoxyribonuclease I (DNase I, 5 units/µL, Takara, Shiga, Japan) were added to the hippocampi in the tube, which were then incubated at 32°C for 12 min. The papain solution was prepared by dissolving 70 mg of papain (0.5 units/g, Wako) and 10 mg of ethylenediamine tetraacetic acid-2Na (Wako) in PBS to a final volume of 20 mL. The papain solution was filtered using a 0.2-µm filter (Sartorius, Gottingen, Germany), divided into 5 mL aliquots, and stored at –30°C. The papain solution was thawed slowly at 4°C for several hours before use.

After incubation and digestion with the enzymes, hippocampi were slowly pipetted using a glass Pasteur pipette 12 times, and then filtered using a wetted cell strainer (40-µm mesh, BD Biosciences) into a 50-mL conical tube. The cell strainer was previously wetted with 10 mL of MEM (Sigma) containing 20% FBS (Gibco) and 1% N2 supplement (100×, Invitrogen, Life Technologies) to prevent nonspecific neuronal cell attachment. The entire hippocampi suspension was poured through the cell strainer, and then 10 mL of 20% FBS/N2/MEM was poured on top to collect neurons remaining on the filter. The total volume of 25 mL was shaken gently to inactivate enzymes, left for 1 min, and then centrifuged at 180×*g* for 10 min. Next, the supernatant was aspirated, and the pellet was resuspended in the culture medium described below at a constant cell density of 3×10^4^ cells/mL. This density corresponded to 8.9×10^3^ cells/cm^2^ in the 12-well multiplates (culture area: 3.38 cm^2^). Subsequently, the cell suspension was plated into the wells of 12-well multiplates in a volume of 1 mL per well. Each well had been previously prepared with 0.5 mL of 25% PuraMatrix, so plating was performed gently. Rat embryonic hippocampal neurons prepared in this way were cultured at 37°C in a humidified atmosphere with 5% CO_2_/95% air.

### Culture medium

The basic culture medium used in the present study was Neurobasal medium (Gibco) containing 2% B27 supplement (50×, Invitrogen) and 0.5 mM L-glutamine (Wako), which is described as medium #0 throughout this article. Extra supplements were added to basic medium #0 as described below, according to the manufacturer’s protocol for Neurobasal medium.

Medium #1: 0.5 mM GlutaMAX I (Invitrogen), which is L-alanyl-L-glutamine and a dipeptide substitute for L-glutamine, was added to medium #0 in place of L-glutamine. Because this supplement breaks down slowly, medium #0 was used as the plating medium, and #1 was used after the first medium change. The protocol for GlutaMAX indicates that it may reduce or remove the need for medium changes. To verify this, we also examined an experimental group wherein the medium was changed only the first three times.

Medium #2: 25 µM L-glutamic acid (Sigma) was added to medium #0 [9]. Medium #2 was used only during plating to prevent toxicity from glutamic acid. After the first medium change, medium #0 was used. In some cases, medium #1 was used after the first medium change, which is described as #2/1.

Medium #3: 25 µM 2-mercaptoethanol (Gibco) was added to medium #0 [24], [25]. This thiol works as a reducing agent, and may replicate *in* *vivo* conditions, where low oxygen causes reduction of many chemical substances.

For comparison with basic medium #0, Dulbecco’s Modified Eagle’s Medium/Ham’s Nutrient Mixture F-12 (DMEM/F12, Invitrogen) and MEM media were also used. DMEM/F12 was supplemented with 0.1% bovine serum albumin (BSA, Sigma), 5 ng/mL brain-derived neurotrophic factor (R&D Systems Inc., Minneapolis, MN, USA), 1% N2 (50×, Invitrogen), and 2% B27 (Invitrogen). MEM was supplemented with 2% FBS and 1% N2 as a low serum medium.

For changes of Neurobasal medium-containing media, mediums #0–#3, half of the medium (0.5 mL per well) was exchanged twice a week. For changes of DMEM/F12 medium, 25% of the medium (0.25 mL per well) was exchanged twice a week. Medium changes were done gently so as not to break the PuraMatrix hydrogel or disrupt neuronal attachment or neurite extension.

### Sandwich culture of primary neurons using PuraMatrix and coverslips

Neuronal cell suspensions prepared as described above were plated on PuraMatrix after equilibration. Thereafter, the neurons were incubated at 37°C in a humidified 5% CO_2_/95% air atmosphere. Approximately 3 h after plating, the neurons had adhered to the PuraMatrix, and were covered with a sterilized round glass coverslip (18 mm in diameter, Matsunami, Osaka, Japan). To determine the best time to cover the cells, three different time points were tested: 1, 3, and 22 h after plating.

### Statistical analysis of neurite length and viability

The suitability of different culture conditions was evaluated based on cell viability and neurite length. Viability is a direct indicator of the possibility of long-term cell culture, and neurite length is an indicator of neuronal growth, differentiation, and maturation. In many studies, neurite length is used as an indicator of normal morphogenesis, gene expression, or molecular dynamics [12], [7], [18], [25], [26], [23].

Neurite length was measured by phase-contrast imaging using a Nikon inverted microscope, mainly with the 20× objective. Neurite lengths were calculated from digital images using their scale bars. Neurite length was measured using a standard technique for measuring the curve length: the neurite curve was approximated by multiple straight line segments, and the lengths of these line segments were added together [27]. For statistical analysis, the five longest neurites in each well were selected [26]. Because it was difficult to trace the lengths of neurites growing on the very rough surface of PuraMatrix, they were excluded from the final analysis. Some neurites overlapped or crossed other neurons or neurites; these overlapping regions were observed at higher magnification (60× objective) to check neurite continuity.

Viability was judged by phase-contrast microscopy using the 20× objective on a Nikon inverted microscope. Viability was calculated from the number of living cells with bright, round, and smooth soma and extended neurites. The initial number of living cells per photographed area was counted directly several hours after plating, or calculated from three values: plating density, well area, and photographed area. The living cell number was counted on each observation day and the ratio of the living cell number to the initial number at plating was used for analysis.

Comparisons between different groups with different culture conditions were performed using Student’s t-tests. The maximum neurite lengths were measured from the five longest neurites in each well on phase-contrast images. Similarly, 5–6 images from different areas in each well were taken to count living cells for cell viability. To study differences in position, three photographs were taken at both the center and the edge of each well.

In order to determine the best culture conditions, experiments were repeated three or more times with different rats or batches of cells. From these replicates, the means and standard errors of the maximum neurite length were calculated once or at several time points during the culture. Similarly, cell viabilities were calculated and compared between different culture conditions.

### Statistical analysis by immunofluorescence staining

Cell viability and neurite length were measured using the LIVE/DEAD Viability/Cytotoxicity Kit (Molecular Probes, Eugene, OR, USA). Fluorescence images were taken using a fluorescence microscope (Ti-S, Nikon) with a triple band filter (4′,6-diamidino-2-phenylindole (DAPI)/fluorescein-5-isothiocyanate (FITC)/Texas red). By counting the numbers of green cells, cell viability was calculated, and by measuring the lengths of green neurites, neurite length was determined. Thus, viability and neurite length were assessed from fluorescence images, as well as from phase-contrast images.

### Immunocytochemistry to determine the neuronal ratio

To distinguish neurons from astrocytes and determine the neuronal ratio, the cells were double-stained with anti-microtubule-associated protein 2 (MAP2) and anti-glial fibrillary acidic protein (GFAP). For immunostaining, cultured cells in a well were washed with 37°C PBS and fixed with 4% paraformaldehyde for 20 min. After washing with 37°C PBS, the fixed cells were permeabilized with 0.1% Triton-X in PBS for 5 min. Following two washes with PBS, the cells were blocked with 10% goat serum in PBS at 37°C for 30 min. Next, the following antibodies were applied: mouse monoclonal anti-MAP2 (1∶50, Sigma) for neurons, and rabbit polyclonal anti-GFAP (1∶400, Dako, Glostrup, Denmark) for astrocytes. The cells were incubated with the primary antibodies in blocking buffer at 37°C for 1–2 h. After washing, the following secondary antibodies were applied: Alexa Fluor 488 goat anti-mouse immunoglobulin G (IgG) (1∶100, Molecular Probes) and Alexa Fluor 594 goat anti-rabbit IgG (1∶100, Molecular Probes). The cells were incubated with the secondary antibodies in blocking buffer at 37°C for 30 min. After washing with PBS and removing the coverslip, if applicable, the cells were mounted with Vectashield mounting medium for fluorescence with DAPI (Vector Laboratories, Burlingame, CA, USA) to counterstain the nuclei. Fluorescence images were obtained using a Nikon Ti-S microscope with a triple band filter (DAPI/FITC/Texas red).

The neuronal ratio (%) was expressed as the percentage of the total cells (i.e. neurons plus astrocytes) made up by neurons. Cell numbers were counted at six positions: three in the center of the well and three at its edge. The total numbers of neurons and astrocytes in each well were used for analysis.

### Synapse activity

To study synapse formation and activity after long-term culture, neurons on PuraMatrix covered by a coverslip were stained with the synaptic vesicle marker FM1-43 (Molecular Probes). A working solution of 20 µg/mL dye was prepared in ice-cold Hank’s balanced salt solution for staining. The culture medium was removed from cultured neurons on PuraMatrix after 49 days *in* *vitro* (DIV), and the staining solution was added to the well. After 1 minute, when recycling synaptic vesicles should have already been stained, the staining solution was removed and the culture medium was replaced to reduce background and non-specific adsorption of the working solution. After removing the coverslip, the stained neurons were observed. Phase contrast images and green fluorescent images were obtained using Ti-S (Nikon) and BZ-9000 (Keyence, Osaka, Japan) microscopes. The BZ-9000 microscope has many specialized functions, such as haze reduction and background adjustment. We used the BZ-9000 microscope to obtain high-resolution images with low background because the PuraMatrix, 3D hydrogel tends to cause non-specific adsorption of dye, high background, low resolution, and unfocused images.

### Ultrastructural analysis of PuraMatrix

To analyze the ultrastructure of PuraMatrix during primary culture, we used a digital microscope (VHX2000/1100, Keyence) that can take 3D optical images non-destructively. It is important to study the surface roughness of PuraMatrix during culture with living neurons in culture medium. We examined PuraMatrix ultrastructure in two different wells using a digital microscope, and obtained similar results. Two kinds of 3D images were examined: color 3D images with 2D cross-sectional profiles, and high contrast 3D images using the high dynamic range function, which is useful for transparent and low contrast surfaces.

### Primary culture without PuraMatrix

To compare culture media under 2D culture conditions without coverslips or PuraMatrix, three kinds of media were used: 2% FBS/N2/MEM, DMEM/F12 medium, and basic medium #0. Primary neurons were plated at 3×10^4^ cells/mL into 2D culture vessels, 35-mm glass bottom dishes (Advanced TC, Greiner Bio-One GmbH, Frickenhausen, Germany), and 12-well multiplates (Advanced TC, Greiner Bio-One). The growth area of these vessels was surface-modified for cell culture, primary cell culture in particular.

## Results

### PC12 cells and PuraMatrix

Before primary neuron culture, the best concentration of PuraMatrix was determined using PC12 cells. When plated on PuraMatrix, PC12 cells adhered within several hours, extended neurites by the next day, and sometimes those neurites extended >1,000 µm. Without PuraMatrix, PC12 cells aggregated in solution, but did not attach to the bottom of the well or extend neurites. The effects of PuraMatrix concentration and cell density on neurite length were studied (Table 1). PC12 cells were plated at three different cell densities, 3×10^2^, 3×10^3^, and 3×10^4^ cells/mL, all of which were tested on PuraMatrix at concentrations of 25%, 50%, and 100%. The maximum neurite length, calculated from the three longest neurites at DIV 23, was 1,000±58 µm (mean ± SE, n = 3) on 25% PuraMatrix, significantly higher (*P*<0.05) than that (around 600 µm) on 50% or 100% PuraMatrix. At 3×10^4^ cells/mL, such long neurites were only rarely distinguishable because the cells had aggregated.

**Table 1:** Summary of the culture conditions tested and the best culture conditions in each case.

ExperimentNo.	Celltype	PuraMatrix	Cell density[cells/mL]	Medium and mediumchange (+/−)*	Coverslipping[h after plating]	Best culture conditionsfor neurite extension overlong distances and maximumneurite length
1	PC12	25/50/100%	3×10^2^, 3×10^3^, 3×10^4^	RPMI 1640/10%horse serum/5%FBS (+), NGF+	–	25% PuraMatrix at 3×10^2^and 3×10^3^ cells/mL,1,000±58 µm (n = 3)
2	E18	25/50%	10^3^, 10^4^, 10^5^	DMEM/F12 medium(+), 2% FBS/N2/MEM (+)	–	25% PuraMatrix,DMEM/F12 medium (+),at 10^4^ cells/mL,340±39 µm (n = 4)
3	E18/E19	25%	3×10^3^, 1–1.25×10^4^, 2.5–3×10^4^, 10^5^	#0 (+/−), DMEM/F12\medium (+/−)	–	#0 (+) at 2.5–3×10^4^ cells/mL,1,600±460 µm (n = 4)
4	E18/E19	25%	2.5–3×10^4^	#0 (+), DMEM/F12medium (+)	–	E18, #0 (+),1,800±586 µm (n = 3)
5	E18	25%	3×10^4^	#0 (+)	−/1/3/22	3 h coverslipping,around 1,800 µm
6	E18	25%	3×10^4^	#0 (+), #1 (+/−),#2 (+), #2/1 (+), #3 (+),DMEM/F12 (+), 2%FBS/N2/MEM (+)	−/3	#0 (+) or #2 (+), 3 hcoverslipping, >2,000 µm
7	E18	None	3×10^4^	#0 (+), DMEM/F12medium (+), 2%FBS/N2/MEM (+)	–	#0 (+), several hundredmicrometers
8	E18	25%/None	3×10^4^	#0 (+)	−/3	25% PuraMatrix,3 h coverslipping,≥3,000 µm

Data are shown as mean ± SEM for sample size n.

The best culture conditions were determined in experiments 1–7, and then a conclusive experiment 8 was performed.

E18, E19: Primary neurons isolated from rat fetal hippocampi on embryonic days 18 and 19.

*medium change (+): medium changes.

*medium change (–): no medium changes.

*For medium name, see “Culture medium” section.

Primary neurons cultured on 25% and 50% PuraMatrix were also compared. The maximum neurite length calculated from the four longest neurites at DIV 3–4 was 340±39 µm (mean ± SE, n = 4) on 25% PuraMatrix, significantly higher (*P*<0.05) than that (188±40 µm) on 50% PuraMatrix. Based on these results and those of a previous study [20], 25% PuraMatrix was selected for all subsequent primary culture experiments.

### Developmental pattern of rat hippocampal primary neurons

**Figure 1:**
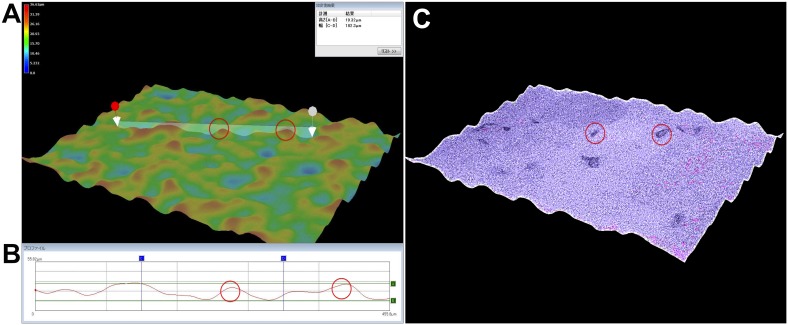
Three-dimensional images of the surface roughness of PuraMatrix with adherent neurons observed using a digital microscope. The neurons were cultured on 25% PuraMatrix in basic medium #0 for 1 month. Two neurons are shown by red circles. (A) Color 3D image. The highest positions are displayed in red and the lowest positions in blue (see color scale at the upper left). The maximum height difference was <40 µm. (B) Cross-sectional profile between the two arrows in A showing a height of 19.32 µm (A–B) and a width of 455.8 µm (C–D: 182.3 µm). The neurons and PuraMatrix show a typical height difference, or surface roughness, of approximately 10–20 µm. (C) High contrast 3D image using the high dynamic range function. The transparent neurons are emphasized by their dark color.

Using our combination method, neuronal cells were sandwiched between the PuraMatrix and a coverslip. Surface roughness, or the variation in height of the 25% PuraMatrix in the culture condition, was determined using the digital microscope (VHX2000/1100, Keyence) to be typically 10–20 µm, and 40 µm at the maximum (Fig. 1).

The effects of the 3D PuraMatrix hydrogel and coverslip on primary cultured neurons were evaluated by measuring neurite length, cell viability, and neuronal ratio. In addition, various cell densities, medium components, medium change, embryonic days for dissociation of the hippocampus, and the timing of the coverslip placement on top of the hydrogel were tested (Table 1). We selected neurite length as a parameter for assessing culture conditions because it is an indicator of whether many neurons survive, differentiate, and mature. Indeed, axons elongate 5–10 times faster than dendrites, and longer neurites are considered to be differentiated axons at maturation stage 5 [12]. We found measurements of spine density or factors other than neurite length to be relatively inaccurate and difficult compared with measuring neurite length because the resolution of neuronal images is decreased by the rough, thick, and semi-opaque 3D PuraMatrix. In addition, it is difficult to achieve proper focus on structures as small as spines on a rough surface. Axons could be measured more simply than any other structure.

**Figure 2:**
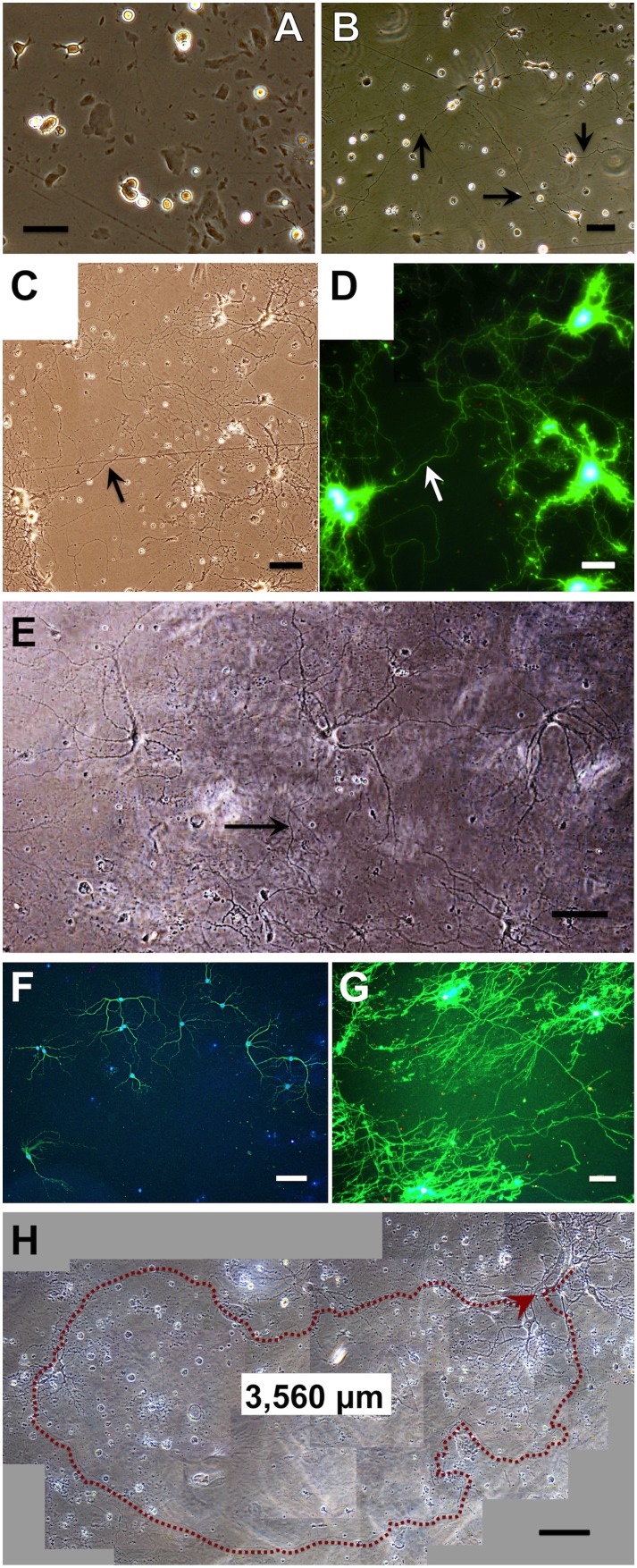
Developmental pattern of primary neurons plated on 25% PuraMatrix at 3×10^4^ cells/mL. The neurons were isolated from the hippocampi of embryonic rats (E18) and cultured in basic medium #0 or #2 (E, H). A coverslip was placed over the neurons 3 h after plating. Bars: 50 µm or 100 µm (E–H). (A) Phase-contrast image of neurons 3 h after plating. The neurons attached to PuraMatrix, and extended lamellae or short neurites within 50 µm. (B) Phase-contrast image of neurons at DIV 1. The neurons started to extend one neurite that was several times longer than the others. The long neurites (arrows, approximately 300 µm) are assumed to be axons, and the other, shorter ones, dendrites. (C) Phase-contrast image of neurons at DIV 21. The neurons extended neurites for several hundred microns (axons, arrow), as well as neurites with branches and spines (dendrites). Axons and dendrites formed fine networks. (D) Fluorescence image of (C), stained with the LIVE/DEAD Viability/Cytotoxicity Kit. Living cells and neurites are stained green, dead cells red. (E) Phase-contrast image of neurons at DIV 36. The neuronal cell bodies are increased in size. Dendrites have characteristic spines that are increased in length, tapered and branched. Axons (arrow) show a uniform diameter over a long distance. The neurons formed neuronal networks and matured. (F) Fluorescence image of neurons at DIV 38. Neurons and astrocytes are double-stained with anti-MAP2 for neurons (green) and anti-GFAP for astrocytes (red), with DAPI as a nuclear counterstain (blue). Neuronal survival for 5 weeks without glial proliferation is shown. (G) Fluorescence image of neurons at DIV 42, stained with the LIVE/DEAD Viability/Cytotoxicity Kit. Neuronal survival and neurite extension is shown. (H) Phase-contrast image of neurons at DIV 63. The neurons show bright, round, and smooth somas, and long axons of a uniform diameter (3,560 µm, arrow), suggesting their survival for 2 months.

The developmental pattern of primary neurons in the best culture conditions as determined in the experiments described below is shown in Figure 2. After plating onto 12-well multiplates prepared with 25% PuraMatrix, the neurons attached and started to extend neurites within 3 h (Fig. 2A), showing characteristics of developmental stages 1 or 2 [12]. Three hours after plating, a coverslip was placed on the neurons in each well. Within several days, the neurons started to extend one neurite that was longer than the others, showing characteristics of developmental stage 3 (Fig. 2B, arrows). Subsequently, the neurons extended these neurites over several hundred microns (now assumed to be axons, arrows in Fig. 2C–D), as well as neurites with branches and spines (dendrites), showing characteristics of developmental stages 4 or 5. Such identification of axons and dendrites based on their length and morphology agrees with immunoreactive staining against MAP2, a protein specific to dendrites [12]. Thus, the neurons differentiated, maturated to developmental stage 5, extended axons (≥3,000 µm), and survived long-term for over 2 months without glial proliferation (Fig. 2E–H). In addition, the neurons continued to maintain fine neuronal networks with a single-cell distribution, i.e., the distances between the neurons were maintained well.

### Assessment of best cell density and medium change

The best cell density and medium change procedures were examined using DMEM/F12 medium and Neurobasal medium/B27/L-glutamine (basic medium #0 as described in “Culture medium” section). Before examining the effects and timing of coverslip placing, we looked at the effects of media type and medium changing, cell density, and embryonic day, because these conditions had greater effects on neuronal growth (i.e., they altered neurite length severalfold).

Media were changed as described in “Culture medium” section (+), or left unchanged (−) for comparison. Cell density was 3×10^3^, 1–1.25×10^4^, 2.5–3×10^4^, or 10^5^ cells/mL. For each condition, the maximum neurite length during culture was measured as an indicator of neuronal survival and active outgrowth without regression. Experiments were repeated three to five times with different rats or batches of cells. The maximum neurite lengths were highest in medium #0 (+) at 2.5–3×10^4^ cells/mL, 1,600±460 µm (mean ± SE, n = 4), although the differences between most groups in medium #0 were not significant. Compared with this condition, neurite lengths in DMEM/F12 medium were significantly shorter (approximately 500 µm, *P*<0.05) for all conditions. Based on these results, the best culture conditions were determined to be a cell density of 3×10^4^ cells/mL (8.9×10^3^ cells/cm^2^) in basic culture medium #0 with medium changes.

### Assessment of embryonic day for isolation of hippocampal neurons

Primary neurons were isolated from rat fetal hippocampi on E18 and E19. Neurons were plated on 25% PuraMatrix at a cell density of 2.5–3×10^4^ cells/mL. Basic medium #0 and DMEM/F12 were used and compared. Neurite length was measured at least once a week, and the maximum value during culture was used for analysis. Experiments were repeated three times with different batches of cells. Neurite length was highest with neurons isolated from E18 hippocampi and cultured in medium #0, 1,800±586 µm (mean ± SE, n = 3), and this length was more than twice that found in E19 hippocampal neurons in medium #0 (833±88 µm). The neurite lengths of cells cultured in DMEM/F12 medium were approximately 500 µm, 490±26 µm for E18 and 497±33 µm for E19 hippocampi. Based on this result, hippocampal neurons from E18 embryos were cultured in medium #0 for all subsequent experiments because this condition provided more than twice the neurite lengths of all the other conditions, though these differences were not significant.

### Timing of coverslip placement

To determine the best timing for coverslip placement on neuronal cells, three different time points were tested: 1, 3, and 22 h after plating of neurons. Cultures without coverslips were also tested. Neurons isolated from E18 embryos were plated on PuraMatrix and cultured in basic culture medium #0 with medium changes as described above. Experiments were repeated three or four times for each time point with cells from different rats. For statistical analysis, means and standard errors of cell viability and maximum neurite length were measured four to six times during culture. Only a few significant differences in cell viability were found among the different coverslip placement times at each measurement point, and that the mean viabilities did not differ either. The maximum neurite lengths were highest with placement at 1 or 3 h when assessed at DIV 3–8, and highest with placement at 3 or 22 h when assessed at DIV 14–38. The maximum neurite lengths with coverslip placement at 3 h were significantly longer than in the other cases (1 h, 22 h, and without a coverslip) at nine time points (DIV 4–35) in all the 4 replicates, and significantly shorter than the 1 h placement at the early two time points (DIV 4, 8) in only one replicate. In addition, long neurites of approximately 1,800 µm were repeatedly obtained only with this coverslip placement timing.

The best time for coverslip placement was chosen as 3 h after plating because 3–5 weeks after plating is an extremely important period for neuronal research, and significantly longer neurites were found during that period only when using 3 h coverslip placement. Based on these results, coverslips were placed on cultured neurons 3 h after plating in all subsequent experiments.

### Comparison of culture media

The best culture medium for primary neurons was determined using the media formulations described in the “Culture medium” section. Primary neurons were suspended in each medium, and then plated into two wells containing PuraMatrix for each medium. In one well for each medium, a coverslip was placed on the cultured neurons 3 h after plating as described above. The condition without a coverslip is hereafter described as medium−, and that with a coverslip is described as medium+. Experiments were repeated three to four times for each medium with cells from different rats. From these repetitions, means and standard errors of the maximum neurite length and cell viability were calculated at 2, 3, 5, and 8 weeks after plating.

The cell viabilities were not significantly different between medium+ and medium– conditions for each medium at 2, 3, 5, or 8 weeks. Cell viabilities in medium #0+ (around 30%) were not different from those in #1+, #2+, or #2/1+, all media composed of Neurobasal medium. However, viabilities in DMEM/F12+ (around 10% or lower) continued to be significantly lower than those in medium #0+.

The maximum neurite length in each medium+ was generally longer than that in each medium–, although there were no significant differences (*P*<0.05), except for #1 at 2 weeks and DMEM/F12 at 5 weeks. The maximum neurite lengths were highest (≥1,000 µm) with conditions #0+ and #2+, which were also longer overall at 3–8 weeks. In addition, extremely long neurites (>2,000 µm) were repeatedly obtained only when using the #0+ and #2+ conditions. However, neurite lengths in #0+ and those in #1+, #2+, or #2/1+ were not significantly different (*P*<0.05), except for #2/1+ at 3 weeks. Neurite lengths in DMEM/F12+ were significantly shorter (around 500 µm) than those in #0+ at 2–8 weeks.

No other media tested produced longer neurites or higher viabilities than media #0 and #2. In particular, low serum medium (2% FBS/N2/MEM) produced extremely short neurites (<100 µm).

Based on these results, #0+ (basic medium #0 with a coverslip) was selected for all subsequent experiments. Although condition #2+ produced a greater mean maximum neurite length, it often resulted in lower cell viability and larger variations in neurite length and viability. The cell viabilities in conditions #0+ and #2+ were 32±2% and 27±3% (mean ± SE, n = 4 for both) at 5 weeks, and 25±4% and 24±5% at 8 weeks, respectively. The maximum neurite lengths in conditions #0+ and #2+ were 1,271±148 µm and 1,374±289 µm at 5 weeks, and 1,032±78 µm and 1,132±345 µm at 8 weeks, respectively. For better stability and reproducibility in long-term culture, basic medium #0 was selected.

Finally, we evaluated the effect of medium alone with neither coverslip nor PuraMatrix. Primary neurons were plated into regular 2D culture vessels at 3×10^4^ cells/mL, and cultured in three different media: 2% FBS/N2/MEM, DMEM/F12 medium, and basic medium #0. When plated onto glass bottom dishes, neurons died or released within several days in 2% FBS/N2/MEM or DMEM/F12 medium. In #0, however, they extended neurites of several hundred microns. When plated into 12-well multiplates, neurons showed similar results, though glial cells proliferated in 2% FBS/N2/MEM. With neither coverslip nor PuraMatrix, #0 achieved higher viability, longer neurites, and decreased proliferation of glial cells.

### Effect of the coverslip

The effect of the coverslip was evaluated using the best culture conditions as described above (see Table 1): primary neurons were plated on 25% PuraMatrix, maintained in basic medium #0, half of the medium was changed twice a week, the cell density was 3×10^4^ cells/mL, the neurons were isolated from E18 rat hippocampi, and a coverslip was placed on the neurons 3 h after plating (+) or not placed for comparison (−).

The experiments over 1–2 months were repeated 14 times with cells from different rats. Based on these repetitions, only a few significant differences in cell viability were found at each time point between coverslip (+) and (−) conditions, and the maximum neurite lengths were significantly longer in coverslip (+) than (−) conditions at one or more time points during DIV 4–60. The maximum neurite length in coverslip (+) condition typically became highest (1,100–1,700 µm) at DIV 24–60 during each repetition. There were significant differences in viabilities between coverslip (+) and (−) conditions at only four times among the 62 time points: viability was higher in coverslip (+) twice, and in coverslip (−) conditions twice as well. Microscopy was mostly performed four to six times to measure cell viability and neurite length, and the resulting values tended to become lower when microscopy was repeated several times before DIV 14. In 4 replicates, microscopy was performed once or not performed before DIV 14, resulting in 39±4% (mean ± SE, n = 4) viability at DIV 35 and 27±5% at DIV 53–60.

**Figure 3:**
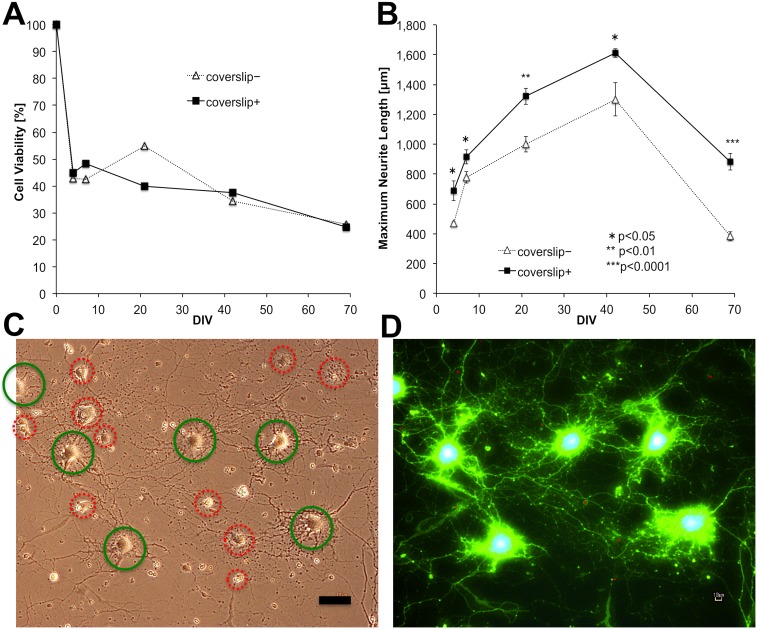
Effect of the coverslip on cell viability and maximum neurite length of primary neurons. Neurons from E18 rat hippocampi were plated on 25% PuraMatrix at 3×10^4^ cells/mL in basic medium #0, and coverslipped 3 h after plating (+), or not (−). The neurons were plated into five multiplates simultaneously, and the cell viability and neurite length in one plate were examined only once at DIV 4, 7, 21, 42, or 69. (A) Cell viability calculated from the mean of two wells. Cell viability was evaluated by phase-contrast microscopy, and is expressed as the fraction of the living cell number 3.5 h after plating (DIV 0, 100%). Living cell numbers were counted in six areas for each well: three areas in the center and three areas at the edge of the well. There were no significant differences between coverslip (+) and (−) conditions in any of the 12 sets of data from the two wells (two-tailed Student’s t-test). (B) The maximum neurite length was expressed as the mean and standard error from the five longest neurites in each well stained with the LIVE/DEAD Viability/Cytotoxicity Kit. For each pair of coverslip (+) and (−) treatments at each DIV, significant differences are shown (one-tailed Student’s t-test). The maximum neurite length in the coverslip+ condition was significantly longer than that in the coverslip− condition throughout the culture period of DIV 4–69. (C) Phase-contrast image of coverslip+ neurons at DIV 21. Bar: 50 µm. (D) Fluorescence image of (C) stained with calcein AM (green) and ethidium homodimer-1 (red). Living cells and neurites are stained green, shown as the bright, smooth cells in C (green circles). Dead cells are stained red, shown as rough or dark cells in C (red circles). C and D show that approximately 40% of the neurons attached to PuraMatrix were alive at DIV 21 and had extended neurites and formed neuronal networks.

To prevent any potential effects of the microscopy and staining on neuron health, the following examinations were performed in 2 of the 14 replicates. Neurons were simultaneously plated into five multiwell plates, and the cell viability and neurite length were examined in one plate at each DIV. The cell viabilities were averaged from two wells as assessed by phase-contrast microscopy, and neurite length was assessed from one well by fluorescence microscopy after staining using a LIVE/DEAD Viability/Cytotoxicity Kit. The viabilities and neurite lengths of these replicates were similar, and one replicate is shown in Figure 3. Cell viability was noticeably decreased within a few days after plating, and then continued to decrease in a linear and extremely gradual manner over DIV 7–69 (Fig. 3A). Viability was 40–50% at 3 weeks, approximately 40% at 6 weeks, and 30% at 2 months after plating. There was no significant difference in viability between conditions (+) and (−). These viabilities were as high as or higher than the 4 replicates mentioned above. At DIV 21, approximately 40% of neurons attached to the PuraMatrix were alive and stained green (Fig. 3D). The average viability at DIV 21 is shown in Figure 3C–D. There was no significant difference between the results determined by phase-contrast and fluorescence microscopy for both cell viability and maximum neurite length (Fig. 3C–D).

**Figure 4:**
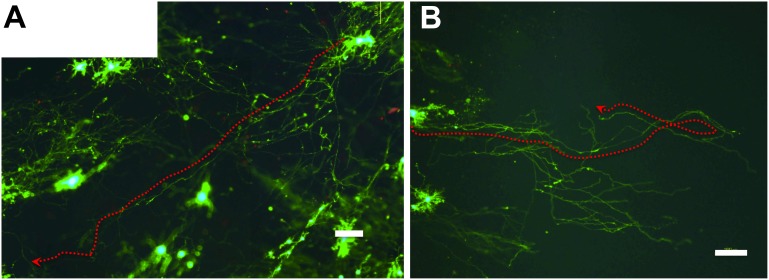
Fluorescence images showing long-term neuronal survival and long neurites at DIV 42 in Fig. 3. The primary neurons were plated on 25% PuraMatrix at 3×10^4^ cells/mL, and coverslipped 3 h after plating (B), or not (A). Living cells and neurites are stained green with calcein AM, and dead cells red (dots) with ethidium homodimer-1. Bars: 100 µm. Both A and B show long neurites (>1,600 µm, arrows). As shown in (A), fluorescence images can be used to study the 3D growth of neurites because neurites on the rough surface of PuraMatrix can be traced by green fluorescence, even if they are out of focus.

The maximum neurite length achieved in coverslip (+) was significantly longer than that in coverslip (−) at all time points examined (Fig. 3B). The difference was extreme at DIV 69 (*P*<0.0001), demonstrating the growth-promoting effect of the coverslip during long-term primary culture over 2 months. Our combination culture with a coverslip and PuraMatrix continued to yield long neurites: >800 µm at DIV 7–69 and >1,300 µm at DIV 21–42 (Figs. 3B, 4). The presence of long neurites (>1,600 µm) at DIV 42 is shown in Figure 4 (note that the density of living cells appears less here than in most of the other images taken to assess viability).

### Neuronal ratio

To evaluate the effects of coverslip and PuraMatrix on the neuronal ratio, we studied four conditions (Table 1, Experiment 8): one with both PuraMatrix and a coverslip (our combination method); one with PuraMatrix but no coverslip; one without PuraMatrix but with a coverslip (conventional sandwich culture); and one with neither PuraMatrix nor a coverslip (regular 2D culture). Here, the neuronal ratio (%) is the ratio of the number of neurons to the total cell number (neurons plus astrocytes) in each well. Neuronal ratio was determined by immunostaining neurons with anti-MAP2 and astrocytes with anti-GFAP. Before plating, PuraMatrix was prepared in only two of the four wells in each multiplate. Experiments with cells from different rats were repeated four times. At DIV 38–42 with PuraMatrix, the neuronal ratio was >90% (92±2%, mean ± SE, n = 4) in the coverslip (+) and 86±3% in the coverslip (−) conditions. Our combination method resulted in higher neuronal ratios than without a coverslip in all the 4 replicates. Without PuraMatrix, the neuronal ratio was 97±2% in the coverslip (+) condition, and lowest (64±9%) in the coverslip (−) condition. Regular 2D culture yielded a significantly lower neuronal ratio (*P*<0.05) than all the other conditions, including our combination method.

**Figure 5:**
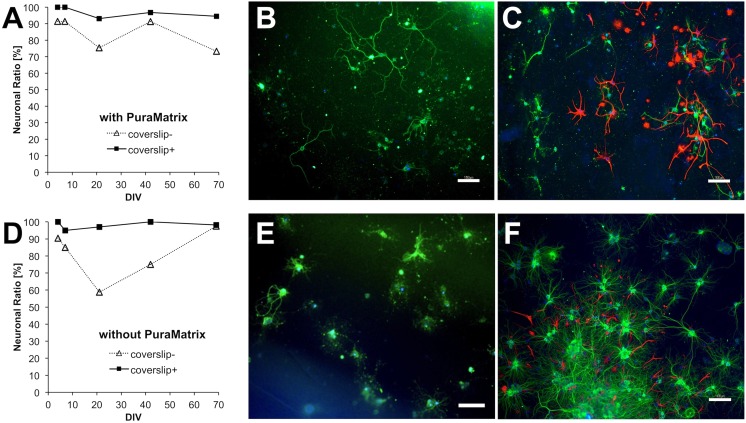
Effect of the coverslip and PuraMatrix on the neuronal ratio of primary neurons. Bars: 100 µm. Neurons from E18 rat hippocampi were plated at 3×10^4^ cells/mL in basic medium #0 and coverslipped 3 h after plating (+), or not (−). Before plating, 25% PuraMatrix was already prepared in a well (A–C) or the wells were not treated (D–F). The neurons attached to PuraMatrix (A–C) or the polystyrene, tissue culture-treated bottom (D–F) and grew. The neurons were plated into five multiplates simultaneously, and the neuronal ratio in each plate was only examined once at DIV 4, 7, 21, 42, or 69. Neurons and astrocytes were double-stained with anti-MAP2 for neurons (green) and anti-GFAP for astrocytes (red), with DAPI as a nuclear counterstain (blue). Neurons and astrocytes were counted in six areas in each well: three areas in the center and three areas at the edge, and their total numbers were used for analysis. The neuronal ratio was consistently higher in the coverslip+ compared with the coverslip– condition at DIV 4–69 (A, D). (A) Our combined sandwich culture technique using both a coverslip and PuraMatrix (coverslip+) achieved a high neuronal ratio of nearly 100%. Fluorescence images at DIV 42 at the edge of the coverslip+ (B) and coverslip− (C) wells. (B) Neurons (green) preceded astrocytes (red). (C) Astrocytes proliferated locally, mainly at the edge of the well. (D) Sandwich culture using coverslip only (coverslip+) also yielded a high neuronal ratio of nearly 100%. Fluorescence images at DIV 42 at the edge of the coverslip+ (E) and in the center of the coverslip− (F) wells. (E) Neurons (green) preceded astrocytes (red). The image is unfocused because of the influence of intrinsic fluorescence of the polystyrene well itself, especially at the edge. (F) Astrocytes proliferated locally, mainly in the center of the well, where many cells aggregated.

Two of the 4 replicates were performed as described above: neurons were simultaneously plated into five multiplates, and the neuronal ratio was examined in one plate at each DIV. The neuronal ratios of these replicates were similar and higher than the other two at DIV 38–42, and one replicate is shown in Figure 5. Our combination method with both PuraMatrix and a coverslip resulted in a neuronal ratio of almost 100% for >2 months, with 97% on average and 95% at DIV 69 (Fig. 5A–B). Using PuraMatrix without a coverslip, the neuronal ratio was slightly, but significantly (*P*<0.05) lower at 85% on average and around 80% after DIV 21 (Fig. 5A, C). The specific growth patterns in each condition are shown in Figure 5B–C, E–F, but these are not representative of the average neuronal ratios and viabilities.

Without PuraMatrix, the neuronal ratio varied depending on the presence or absence of a coverslip. With a coverslip, the neuronal ratio was 98% on average. Although this neuronal ratio was slightly, but not significantly higher than our combination method, the neurites were shorter, indicating their depression or regression (Fig. 5D–E). Without a coverslip, the neuronal ratio decreased at first, and then increased again to be 81% on average (Fig. 5D, F). This increase at DIV 42–69 might have occurred because many astrocytes died and detached from the well as the number of DIV increased, while the neurites of the remaining neurons regressed.

In the absence of a coverslip, astrocytes proliferated locally, mainly at the edge of the wells with PuraMatrix (Fig. 5C), and mainly in the center of the wells without PuraMatrix, where both the neurons and astrocytes aggregated (Fig. 5F).

### Position within the well

To compare neuronal survival between the center and edge of the well, we calculated the cell viabilities at both positions. Experiments were repeated three times with cells from different rats. With PuraMatrix and a coverslip, viabilities were around 50% in the centers of wells until DIV 35–42; without PuraMatrix but with a coverslip, viabilities were low (20–30%) at DIV 7–69; and without either PuraMatrix or a coverslip, viabilities were several times higher in the center than at the edge because of cell aggregation and glial proliferation. Our combination method with PuraMatrix and a coverslip produced consistent viabilities of around 50% in the centers of wells for 5–6 weeks that were higher than in all other conditions, except for the condition with neither PuraMatrix nor a coverslip. In cultures without PuraMatrix, the difference in cell viabilities between positions was larger, and the viabilities in the center of wells were often significantly higher than at the edge.

### Examples of longer neurites

**Figure 6:**
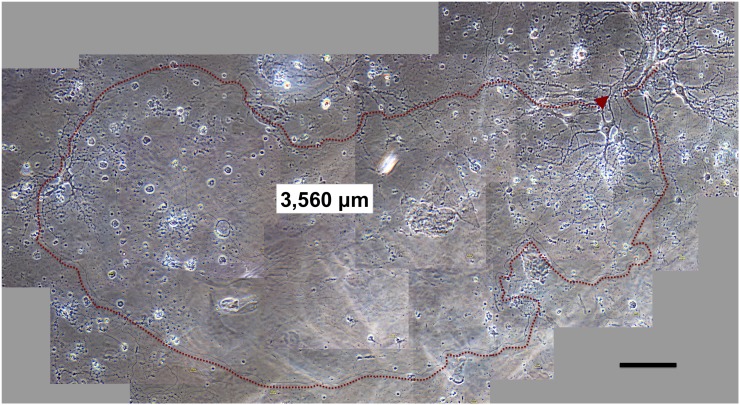
Phase-contrast image of a neuron extending a long neurite (>3,000 µ**m) at DIV 63.** Bar: 100 µm. Neurons from E18 rat hippocampi were plated at 3×10^4^ cells/mL in medium #2, and coverslipped 3 h after plating. The neurons show bright, round, and smooth somas, and a characteristic morphology of tapered dendrites with spines and long axons with a uniform diameter (3,560 µm, arrow), suggesting they could survive after the long-term culture for 2 months. Continuity of the axon was confirmed by high-powered microscopy, especially at intersection points of this axon and another neurites (upper) or neurons (left).

The extremely long neurites obtained with coverslips and PuraMatrix occasionally reached 3,000 µm in basic medium #0 and medium #2. An example neurite of 3,560 µm observed at DIV 63 in medium #2 with a coverslip is shown in Figure 6. This image was selected to show the presence of long neurites, even though the number of living cells in this figure is less than that in the images showing average viability.

Even without a coverslip, neurites of around 3,000 µm were sometimes observed when they migrated into a gap between the two layers of PuraMatrix (e.g. 2,900 µm in #0 at DIV 22). The lower layer of PuraMatrix formed the culture substratum, and another small layer overlapped it.

### Synaptic activity

**Figure 7:**
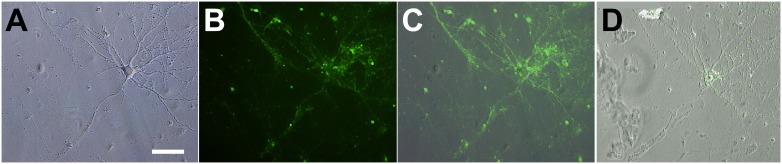
FM1–43 staining of primary neurons at DIV 49. Imaging was performed using the Ti-S (Nikon) (A–C) and BZ-9000 (Keyence) (D) microscopes. Neurons from E18 rat hippocampi were plated at 3×10^4^ cells/mL in basic medium #0 on 25% PuraMatrix and coverslipped 3 h after plating. Bar: 50 µm. (A) Phase-contrast image of one neuron. The neuron shows a bright, round, and smooth soma, indicating its viability after long-term culture for 7 weeks. (B) Green fluorescence image of (A), showing synaptic activity. (C) Merged image of the phase-contrast (A) and fluorescence (B) images. (D) Merged image of the phase-contrast and fluorescence images of the same neuron taken using the BZ-9000 microscope. The ultrastructure of the micron-sized spots on the neuron are distinct, indicating synaptic activity.

We examined synaptic activity and functional maturation of primary neurons using the endocytotic marker FM1-43 at DIV 49 using two different microscopes, the Ti-S (Nikon) and BZ-9000 (Keyence). Phase-contrast images obtained using the Ti-S microscope (Fig. 7A) indicated neuronal survival, but their ultrastructures, as stained with FM1-43, were unclear (Fig. 7B–C). However, images taken using the BZ-9000 microscope were distinct, and stained puncta on the order of microns could be observed on the neurons (Fig. 7D), demonstrating that active synaptic vesicle recycling was occurring after long-term culture for 7 weeks.

## Discussion

Our combination method of the sandwich culture technique using PuraMatrix and coverslips provided the following advantages: (1) long-term culture of primary neurons on a 3D hydrogel without glial feeders for over 2 months at a low density of 3×10^4^ cells/mL (8.9×10^3^ cells/cm^2^); (2) longer neurites (≥3,000 µm) than in culture conditions without a coverslip; (3) an almost pure culture of neurons with an average neuronal ratio of 97%; (4) cell viability ≥40% for 6 weeks and ≥30% for 2 months; and (5) a uniform, single-cell distribution of neurons without aggregation.

### Long-term culture on 3D hydrogel without glial feeders at a low density

Our studies suggest the possibility of longer culture periods or lower cell densities without glial feeder layers than previous studies using 2D sandwich culture, including sandwich co-culture with glial cells (<1 month) [4], [8], [10], [1], [3], [5], [6], [7], [13]. Because our experimental system does not involve a glial feeder, it can be created more simply within a shorter period of several hours, and the evaluation and interpretation of the obtained results is simpler. In addition, our culture system using a 3D nanofibrous hydrogel reproduces *in* *vivo*-like conditions. 2D culture resulted in undesirable effects (Fig. 5D–F), including neurite depression and low cell viability using a coverslip, and cell aggregation and glial proliferation without a coverslip. These results may be partially due to the use of tissue culture-treated multiplates with no ECM coating. However, our culture system using a 3D nanofibrous hydrogel provided better results than those obtained in 2D cultures both in this study and in previous studies cited above. Furthermore, our methods may eliminate the need for a coating procedure, as well as the need for glial feeder layers.

Neurite length continued to increase until DIV 42 (Figs. 3B, 4), suggesting that our culture methods were able to suppress regression of axons for 6 weeks. In addition, synaptic activity at DIV 49 was demonstrated (Fig. 7). Namely, our culture methods can maintain axonal growth, neuronal function, and synaptic activity for at least 6–7 weeks.

### Longer neurites than in culture conditions without a coverslip

The maximum neurite length was longer with a coverslip than without (Fig. 3), suggesting that the coverslip continued to assist neurite extension during long-term culture >2 months. The longest neurites of primary neurons observed here extended 3,000 µm (Fig. 6). These neurites are 10 times [12] or four times [7] longer than those seen in studies using glial feeders, and 10 times [18] or four times [28] longer than those seen in studies using PuraMatrix [18] or polyacrylamide gels [28].

Even without a coverslip, long neurites of around 3,000 µm were sometimes observed to migrate between the two layers of PuraMatrix. In such cases, the upper layer of PuraMatrix may have acted like a coverslip by creating low-oxygen conditions.

The longest neurites stained with MAP2, which were assumed to be dendrites, were approximately 300 µm (Fig. 5B). Therefore, the longest axons were about 10 times longer than the longest dendrites. This result agrees with a previous report [12] and demonstrates that the growth rate of axons was 5–10 times greater than that of dendrites.

### Nearly pure neuronal culture without glial proliferation

Our combination method produced a consistently high neuronal ratio without astrocytes for over 2 months as shown in Figure 5, significantly higher than without a coverslip. Our results indicated that the effects of the coverslip included the promotion of neuronal growth, suppression of glial proliferation, enhancement of neuronal ratio, and creation of a nearly pure culture for the long term. Since glial proliferation is undesirable in many neuronal cultures, our methods may be greatly beneficial for other neuroscience studies.

The suppression of astrocyte proliferation by PuraMatrix or coverslip placement was indicated by the results of 4 replicates at DIV 38–42 showing that the neuronal ratio for cells with only PuraMatrix and only a coverslip were both significantly higher than that with neither coverslip nor PuraMatrix. Glial proliferation with neither PuraMatrix nor a coverslip is in agreement with previous studies, which showed that neurons grew well on collagen [23] or polyacrylamide [28] gels, whereas glia proliferated on 2D coated surfaces.

A sandwich culture method using coverslips and cytosine arabinoside has been proposed [10]. The neuronal ratio achieved with this method was 95% at DIV 5 at a density of 10^5^ cells/mL. However, those cultures showed short-term survival at high density, and cytosine arabinoside has toxic effects. In this regard, our sandwich culture method is favorable because it can reproduce *in* *vivo*-like low-oxygen conditions without a toxic substance.

### Cell viability greater than 30% for 2 months

Cell viability of ≥30% for 2 months or ≥40% for 6 weeks was not significantly different from that without a coverslip, as shown in Fig. 3, suggesting that our sandwich culture method did not decrease neuronal survival. The viability decreased to approximately 50% within several days, but was still higher than that reported in previous studies with cells at the same low densities as ours [4], [5], [6], [7], [9], [10]. After that time, our methods provided an extremely slow decrease in viability for >2 months, which differs from previous studies.

Although the viability of cells in our study was relatively low (<50%), it remained higher than that reported in previous studies. Because neurons cultured using our method exhibited normal development (Fig. 2), long-term axonal elongation (≥3,000 µm), synaptic activity at DIV 49, and survival of many neurons without astrocytes, this technique represents an advance over existing culture methods and provides a system for creating *in* *vivo-*like culture conditions.

The cell viability shown in Figure 3A was as great or greater than that in the 4 replicates that underwent repeated microscopy and in the “Comparison of culture media” section, possibly because microscopy was performed only once in the experiment shown in Figure 3, but several times in the other experiments. Microscopy has an adverse effect on cultured neurons owing to the optical illumination and change in temperature and CO_2_ concentration. Based on the 12 replicates, microscopy before DIV 14 appeared to be especially harmful.

Likewise, the maximum neurite lengths shown in Figure 3B were longer than those in the “Comparison of culture media” section, and neuronal ratios shown in Figure 5 were highest in the 4 replicates, probably because of the same reasons. Thus, we believe the results shown in Figs. 3–5 are the most accurate.

### Uniform, single-cell distribution of neurons

The combination approach also provided a uniform distribution of primary neurons and high viabilities in the center of the well (around 50% for 5–6 weeks). Furthermore, a near single-cell distribution was achieved, and unfavorable aggregation was suppressed over the long term (>2 months, Fig. 2). This culture condition of a uniform, single-cell distribution will be greatly advantageous for long-term research of individual neurons and neuronal networks.

In a previous study [4], most neurons sandwiched by a coverslip survived along its edge, probably because the oxygen in the center was too low. In contrast, our combination method provided a uniform distribution across the well, possibly because of the ultrastructure of PuraMatrix. That is, the fibrous nanostructure and rough surface, with a maximum height difference of <40 µm (Fig. 1), may have improved the microenvironment and the microgradients of oxygen, paracrine factors, and other chemical substances.

### Synaptic activity

After long-term culture for 7 weeks using our culture methods, synaptic vesicle turnover was detected by FM1-43 staining, as shown in Figure 7. Stained spots of 1–2 µm indicate the presence of active presynaptic terminals, suggesting that our methods were able to generate and maintain active, functional synapses over the long term. Extremely small spots were visualized using the BZ-9000 microscope as discussed below.

### Other culture conditions

Basic medium #0 provided much longer neurites (>1,600 µm) than DMEM/F12 (around 500 µm) and 2% FBS/N2/MEM (<100 µm) (Table 1). The comparable supporting effects of media #0, #1, #2, and #2/1 on neuronal outgrowth seem to be due to the common components, Neurobasal medium and B27, rather than L-glutamine, glutamic acid, or GlutaMAX. Based on the results for conditions with neither a coverslip nor PuraMatrix, basic medium #0 had the same beneficial effects as coverslips and PuraMatrix. Combining all components of our method (coverslips, PuraMatrix, and culture medium) should be used for best results.

Our finding that 25% PuraMatrix resulted in significantly longer neurites (1.7–1.8-fold) in PC12 and primary neurons than 50% or 100% suggests that it promotes neuronal differentiation, which is consistent with the results of previous studies [20], [28].

In addition, we found that the earlier a coverslip was placed, the faster neurons extended neurites and the faster they regressed, with no effect on cell viability. Few previous studies have compared different coverslip placement or inverting time points, but 1 h [4], [8], 1.5 h [10], 3–4 h (the same as our study) [1], [3], and 24 h [29] after plating have been reported.

Regarding the embryonic day of isolation of hippocampal neurons, E18 rat hippocampal neurons developed neurites of more than twice the length of those developed by E19 neurons in #0, in agreement with the general principle that the younger an organism is, the higher its regenerative capacity.

Microscopy was sometimes repeated, and the same neurites may therefore have been measured on different days. However, this likely did not affect the comparisons of culture conditions. If culture conditions were suboptimal, neurites that initially extended would regress and become shorter at earlier times. The extension of neurites over the long-term indicated that the culture conditions were appropriate. In the experiments shown in Figures 3–5, where microscopy was only performed once, the benefits of the coverslip+ over the coverslip– condition were even greater than in the other experiments with repeated microscopy. In addition, we performed unpaired t-tests to assess the significance of differences in neurite length and cell viability between different groups, such as coverslip+ and coverslip–; these two groups were independent and did not include the same neurites that may be measured repeatedly.

### 3D culture imaging

Image quality in our culture system is decreased by the rough, thick, and semi-opaque 3D substrates. In addition, it is difficult to focus on cells or neurites extending three-dimensionally on the rough surface of 3D substrates. Furthermore, dyes tend to be adsorbed by 3D substrates non-specifically, raising the fluorescent background. If 3D substrates are washed too many times to reduce background noise, they can lose mechanical integrity and soft substrates, as used here, may collapse.

In our studies, the phase-contrast and fluorescence images (Figs. 2, 3C, 5, 6, 7A–C) might be unfocused or have high-background compared with those in 2D culture. We used the LIVE/DEAD Viability/Cytotoxicity Kit (Figs. 2D, 2G, 3D, 4) because neurons could be stained clearly after a single-step assay, and even neurites extending three-dimensionally on PuraMatrix could be traced (Fig. 4A).

We used a BZ-9000 microscope after staining with FM1–43. Although synaptic vesicles are 1–2 µm in size and difficult to identify using a normal microscope (Fig. 7A–C), we were able to take high-resolution images using the BZ-9000 microscope without any preprocessing or complicated operations (Fig. 7D).

We also used a digital microscope to analyze the surface roughness of PuraMatrix in our culture conditions. Although SEM or TEM is often used for 3D analysis, preprocessing is required, and the images may contain artifacts from those steps different from those in culture conditions. With other non-destructive optical 3D microscopes, measuring transparent surfaces like PuraMatrix with neurons in medium is difficult. However, by using a digital microscope (VHX2000/1100, Keyence), we were able to determine the surface roughness of PuraMatrix as shown in Figure 1. These microscopes and fluorescence dyes have good potential for use in 3D culture imaging and analysis.

### Applicability and limitations

Because our culture system was capable of producing cells with long axons (≥3,000 µm), it may prove useful as an *in* *vitro* culture system for studying axonal regeneration of CNS neurons *in* *vivo*. As shown in Figure 5, glial proliferation was also suppressed, suggesting the possibility of suppressing glial scar formation, which is thought to be one of the factors that inhibit the regeneration of CNS neurons. Namely, our culture system has the potential to be a significant advance in regenerative medicine and tissue engineering because CNS neuronal regeneration *in* *vivo* following nerve injury (traumatic brain injury or spinal cord injury) is extremely poor and motor functional recovery is limited.

Several limitations of the present study should be discussed. Only hippocampal neurons and PC12 cells from rats were used in this study. To strengthen the benefits and applicability of our method, it should be applied to various other types of cells, such as motor neurons, and for 3D differentiation culture of induced pluripotent stem cells or embryonic stem cells. Regarding the maximum neurite length, we did not measure all neurites, but subjectively selected long neurites. However, long neurites were typically observed in limited areas of the well where neurons were sparse. Fluorescence images also helped us detect long neurites. It would be very difficult to measure the lengths of all neurites because they overlap and exist in 3D. However, comparisons of neurite lengths measured by the same methods would not likely affect the results showing the benefits of our culture methods over other conditions.

## Conclusions

Our combination approach, using 25% PuraMatrix and coverslips, with the medium and timing parameters described above, provided the best results for the long term, nearly pure culture of rat hippocampal neurons. These techniques promoted neuronal survival and neurite extension, suppressed glial proliferation and cell aggregation, and reproduced *in* *vivo*-like culture conditions.
